# Can information infrastructure development improve the health care environment? Evidence from China

**DOI:** 10.3389/fpubh.2022.987391

**Published:** 2022-08-25

**Authors:** Chenglin Tu, Chuanxiang Zang, Yuanfang Tan, Yu Zhou, Chenyang Yu

**Affiliations:** ^1^Academy of Guangzhou Development, Guangzhou University, Guangzhou, China; ^2^School of Management, Guangzhou University, Guangzhou, China

**Keywords:** information infrastructure, health care environment, spatial difference-in-difference model, spatial spillover effects, mechanism analysis

## Abstract

Existing studies ignore the importance of information infrastructure development in improving regional health care environment. This paper adopts a spatial difference-in-difference (DID) model to assess the impact of information infrastructure development on urban health care environment based on a quasi-natural experiment of the “Broadband China” city pilots (BCCP). A balanced panel of 259 cities from 2010 to 2019 is selected for empirical analysis in this paper. Our findings show that the implementation of BCCP resulted in a 4.1 and 2.9% improvement in local medical workforce and medical infrastructure. In addition, there is significant spatial spillover effects of the implementation of BCCP, with 7.2 and 12.5% improvement in medical workforce and medical infrastructure in the surrounding areas. Our findings also suggest that information infrastructure development enhances the health care environment by driving industrial upgrading and education levels. Further analysis shows that BCCP has the strongest improvement on medical workforce in the eastern region and non-ordinary prefecture-level cities. For medical infrastructure, BCCP has stronger improvement in central region, western region, and non-ordinary prefecture-level cities. Finally, the paper conducts a series of robustness tests to ensure the reliability of the analysis results, including parallel trend tests, placebo tests, and re-estimation with different methods. Policies to improve the health care environment through information infrastructure development are proposed.

## Introduction

With the emergence of environmental pollution and food problems, people are paying more attention to their own health. Improving the health care environment is considered an important means of ensuring public health (1). A large number of scholars have paid attention to how to improve regional health care environment. Swift (2) pointed out that the level of regional economic development is an important factor affecting the health care environment. Population size and structure are also thought to significantly impact the health care environment (3). In addition, some scholars proposed that foreign investment, government intervention and financial development are all essential for the improvement of health care environment (4–8). However, the marginal benefits of these factors are diminishing and there is an urgent need to find new driving factors to further enhance the health care environment.

The development of information communication technology such as big data, cloud computing and 5G provides new opportunities for the improvement of the health care environment (9–11). These technology applications are extremely dependent on government support for information infrastructure (12). On the one hand, the development of information infrastructure has stimulated the application of information technology in the medical field (13). This has greatly enhanced medical cooperation between different regions and can effectively improve the health care environment in developing regions. On the other hand, the development of information infrastructure can promote industrial upgrading and provide talents and technologies for medical institutions, thus improving health care environment (14). This means that information infrastructure development has a significant impact on the health care environment. Especially in the current context of rapid development of information technology, ignoring the impact of information infrastructure construction on the healthcare environment is not conducive to maintaining public health. Therefore, it is necessary to explore the effects and mechanisms of information infrastructure development on the health care environment and propose the recommendations to improve the health care environment.

To develop information infrastructure, China launched a “Broadband China” city pilots (BCCP) policy in 2014. The implementation of BCCP is dedicated to improving the speed and accessibility of broadband, which can effectively improve the level of information technology in the area where it is located. From 2014 to 2016, 116 city pilots have been approved (15). Based on the above quasi-natural experiment, this paper adopts a spatial difference-in-difference (DID) model to assess the impact of information infrastructure development on the health care environment. In addition, this paper further explores the mechanisms by which information infrastructure development enhances the health care environment from the perspectives of industrial upgrading and the education level. Finally, this paper conducts heterogeneity analysis and robustness test.

The contributions of this paper are mainly reflected in the following three points. First, this paper correlates information infrastructure with the health care environment. To the best of our knowledge, there are no studies that analyze the impact of information infrastructure development on the health care environment at the city level. Information infrastructure development greatly enhances the digital health care technology and information health care talent clustering in the region. Neglecting the development of information infrastructure is not conducive to effective improvement of the health care environment. Second, this paper assesses the impact of information infrastructure development on the health care environment through a quasi-natural experiment of BCCP. This policy has a strong exogeneity, which can reduce the interference of endogeneity on the evaluation results to a certain extent. Finally, this paper considers spatial factor to the traditional DID model. There is a clear spatial correlation in China's health care environment, i.e., the improvement of the health care environment in large cities has a spillover effect on the surrounding areas (16). Ignoring such spatial correlation may lead to biased estimates. The results estimated by the spatial DID model reduce the interference of spatial factors and can more reliably assess the impact of political information infrastructure development on the health care environment.

The reminder of this study is organized as follows: Section Research hypothesis provides the research hypothesis of this study, Section Policy background of information infrastructure development of “Broadband China” provides the policy background of information infrastructure development of “Broadband China,” Section Methods and data provides methods and data, Section Results the results, and Section Conclusion and recommendations provides the conclusions and recommendations.

## Research hypothesis

It has been widely recognized in the literature that information infrastructure development has a significant enhancing effect on economic development in terms of productivity enhancement (17–19). However, the impact of information infrastructure development on the health care environment has not been emphasized. This paper argues that information infrastructure development affects the healthcare environment through the following two main channels.

First, the development of information infrastructure has greatly contributed to the upgrading of the industry and provided more advanced collaboration models for health care organizations (20). The mobility of medical technology and personnel is the most important factor limiting the improvement of the health care environment (21, 22). The development of information communication technology represented by 5G and cloud computing can effectively link medical institutions in developed and developing regions, thus enhancing the medical and health care environment in developing regions (16, 23, 24). In addition, the industrial upgrading driven by the development of information infrastructure has also gathered more medical professionals for cities, which is conducive to improving the local health care environment (25).

Second, the development of information infrastructure lowers the barriers to education, and the level of education of residents is improved. On the one hand, this will lead to residents paying more attention to their own health, thus enhancing government investment in health care, and improving the health care environment (26). On the other hand, the improvement of education level will also help to cultivate medical professionals and improve the medical system, which will lead to the improvement of the medical and health care environment (27). Thus, this paper proposes the following hypothesis:

**H1:** Information infrastructure development can improve the health care environment, and its main mechanism is to promote industrial upgrading and education level.

Since the construction of information infrastructure is not divided by administrative divisions, neighboring cities are able to share part of the information infrastructure (28). Information infrastructure development not only affects the local health care environment, but also has an impact on the surrounding areas. On the one hand, the information infrastructure construction improves the information level of local medical institutions, and the neighboring areas will learn and imitate the introduction of advanced technology to improve their own health care environment (29). On the other hand, the information technology and medical professionals gathered by the local information infrastructure construction also provide a reservoir of human resources for the surrounding areas (30). This will lead to the introduction of more medical talents in the surrounding areas, thus improving the medical and health care environment. Therefore, this paper proposes the following hypothesis:

**H2:** Information infrastructure development not only enhances the local health care environment, but also has positive spillover effects on neighboring areas.

## Policy background of information infrastructure development of “Broadband China”

**Figure 1:**
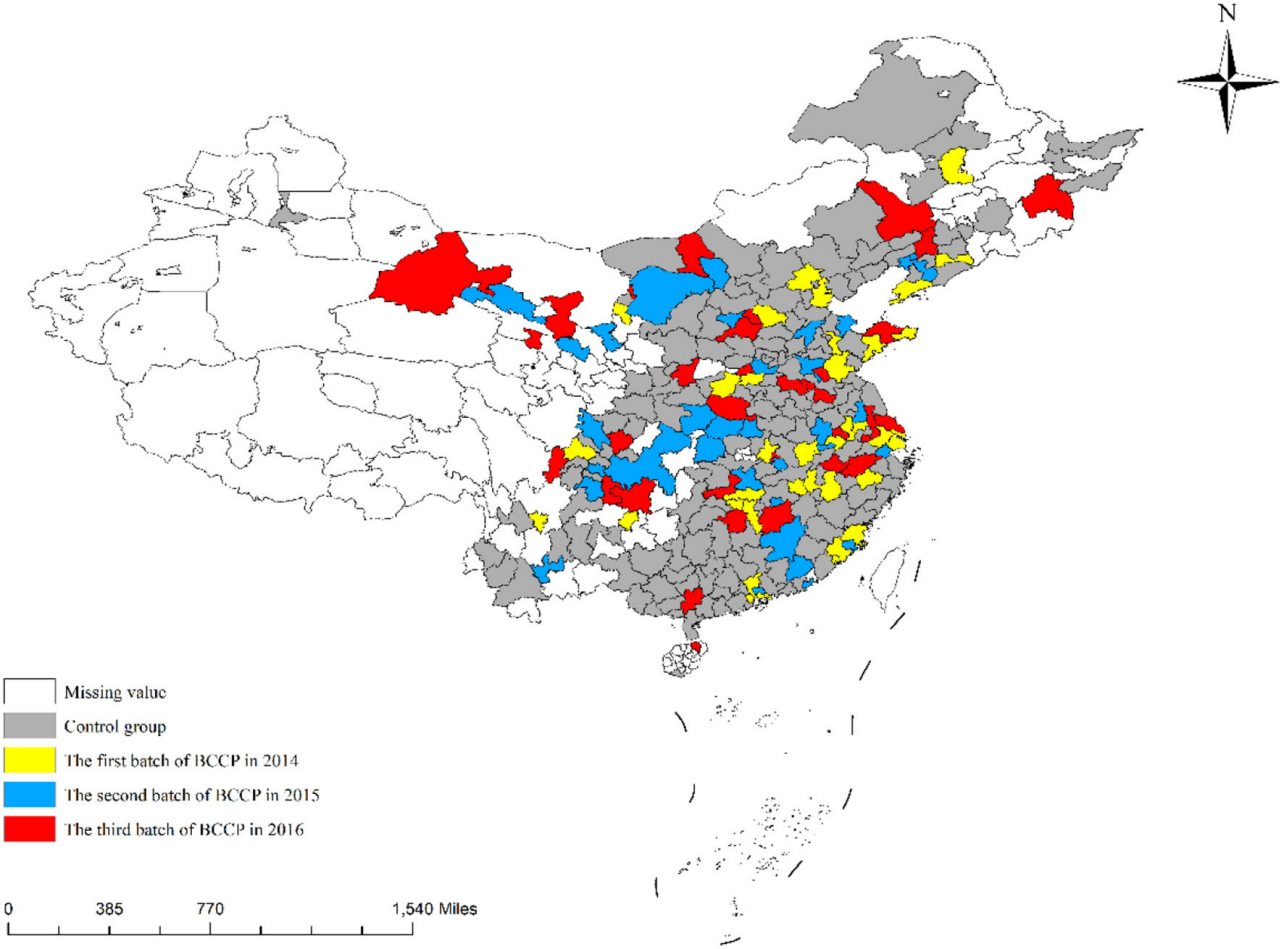
Spatial distribution of the implementation of BCCP.

The development of big data, cloud computing and 5G technologies has further enhanced the transmission and use of information and has had a significant socio-economic impact (28, 31). However, the development and application of these technologies are extremely dependent on the construction of information infrastructure, such as the laying of communication base stations and fiber optic cables (15). Chinese government implemented the policy of “Broadband China” city pilots (BCCP) in August 2013. This policy is dedicated to accelerating the construction of network bandwidth in pilot cities and improving the speed and accessibility of broadband in pilot cities. The first batch of 41 cities with comprehensive expert assessment was implemented in 2014, the second batch of 38 cities in 2015, and the third batch of 37 cities in 2016, the spatial distribution of each city is shown in Figure 1. The main way of the policy implementation is to expand the access coverage to build the network with broadband and promote industrial optimization and upgrading and economic transformation with more diversified network applications. Undoubtedly, the implementation of BCCP has greatly promoted the development of information infrastructure in the pilot cities.

## Methods and data

### Data

The period of the sample in this paper is from 2010 to 2019, covering the first 4 years and the last 5 years of BCCP implementation. Data were obtained from the China City Statistics Database (CCSD) in the China Research Data Service (CNRDS) platform (https://www.cnrds.com/Home/Index#/FinanceDatabase/DB/CCSD) and the China City Statistics Yearbook (2011–2020). Since the spatial DID model requires the data structure to be a balanced panel, this paper excludes the sample of cities that have missing values in any year. The balanced panel dataset contains 259 cities per year, with a total of 2,590 samples.

### Empirical model

#### Baseline model

This paper adopts BCCP as a quasi-natural experiment to evaluate the impact of digital infrastructure development on the health care environment. In addition, considering the existence of spatial spillover effects on the healthcare environment, this paper further adds spatial factors to the traditional DID model and uses spatial DID to assess the impact of BCCP implementation on the health care environment. The estimation results of spatial lag model (SLM), spatial error model (SEM) and spatial Durbin model (SDM) are also reported in this paper. Among them, the SLM incorporates the spatial lag term of the dependent variable in the model (32, 33). The SLM-based spatial DID model is constructed as follows:


(1) $$\begin{array}{ll} HCE_{it}=\alpha +\delta \sum_{j=1}^{n}W_{ij}HCE_{it}+\beta {BCCP}_{it}+\lambda {Control}_{it}\\ + & \varepsilon_{it},\;\varepsilon_{it}\sim N\left(0,\sigma^{2}I\right) \end{array}$$

where *HCE*_*it*_ denotes health care environment of city *i* in year *t*, *BCCP*_*it*_ denotes implementation of the “Broadband China” city pilots, $\delta \overset{n}{\underset{j=1}{\sum }}W_{ij}HCE_{it}$ denotes the spatial lag of c health care environment, *Con*_*it*_ denotes the control variables. The SEM incorporates the spatial lag term of the error term into the model (34), and the spatial DID model based on SEM is constructed as follows:


(2) $$\begin{array}{l} HCE_{it}=\alpha +\beta {BCCP}_{it}+\lambda {Control}_{it}+\varepsilon_{it} \end{array}$$

(3) $$\begin{array}{l} \varepsilon_{it}=\delta W_{it}\varepsilon +\mu ,\;\mu \sim N\left(0,\sigma^{2}I\right) \end{array}$$

where *HCE*_*it*_ denotes health care environment, *BCCP*_*it*_ denotes implementation of the “Broadband China” city pilots, *Con*_*it*_ denotes the control variables, δ denotes the estimated coefficient of the spatial auto-correlation error term; μ denotes the error term. The SDM incorporates the spatial lagged terms of both the independent and dependent variables into the model (35, 36), and the spatial DID model based on SDM is constructed as follows:


(4) $$\begin{array}{ll} HCE_{it}=\alpha +\delta \sum_{j=1}^{n}W_{ij}HCE_{it}+\beta {BCCP}_{it}+\\ + & \xi \sum_{j=1}^{n}W_{ij}BCCP_{it}+\lambda Con_{it} \end{array}$$

(5) $$\begin{array}{l} +\tau \sum_{j=1}^{n}W_{ij}Con_{it}+\varepsilon_{it},\varepsilon_{it}\sim N\left(0,\sigma^{2}I\right) \end{array}$$

where *HCE*_*it*_ denotes health care environment, *BCCP*_*it*_ denotes the implementation of the “Broadband China” city pilots, *Con*_*it*_ denotes the control variables, $\overset{n}{\underset{j=1}{\sum }}W_{ij}HCE_{it}$ denotes the spatial lag term of health care environment, $\overset{n}{\underset{j=1}{\sum }}W_{ij}Con_{it}$ is the spatial lag term of control variables; $\overset{n}{\underset{j=1}{\sum }}W_{ij}BCCP_{it}$ is the spatial lag term of implementation of the BCCP.

#### Mediating effect model

To explore the mechanism of the influence of information infrastructure development on the health care environment, the following mediating effect model is constructed in this paper:


(6) $$\begin{array}{l} HCE_{it}=\alpha +\beta {BCCP}_{it}+\lambda {Control}_{it}+\varepsilon_{it} \end{array}$$

(7) $$\begin{array}{l} M_{it}=\alpha +\beta {BCCP}_{it}+\varepsilon_{it} \end{array}$$

(8) $$\begin{array}{l} HCE_{it}=\alpha +\beta {BCCP}_{it}+\gamma M_{it}+\lambda {Control}_{it}+\varepsilon_{it} \end{array}$$

where ***M***_***it***_ denotes the mediating variable, including industrial upgrading and education level. Industrial upgrading is measured by the ratio of the added value of the tertiary industry to GDP and education level is measured by the number of university students per 10,000 people. If the coefficients of ***BCCP*** in Equation (6) and ***M*** in Equation (7) pass the significance test, it indicates that information infrastructure development affects health care environment through promoting industrial upgrading and education level.

### Variables

The dependent variable in this paper is the health care environment. In this paper, medical workforce and medical infrastructure are selected as the proxy variables for the health care environment (6). Where medical workforce is measured by the total number of doctors and medical infrastructure is measured by the number of hospital beds (6).

The independent variable the implementation of BCCP. BCCP is an incremental reform with three cohorts of cities implementing BCCP from 2014 to 2016. This study uses the implementation of BCCP as the independent variable to assess the impact of information infrastructure development on the healthcare environment. The value of BCCP is 1 if the *i*-th city implemented BCCP in year t and 0 if no BCCP was implemented.

In this paper, a series of factors that determine the urban health care environment are also selected as control variables, including economic development (ln***rgdp***), total population (ln***pop***), foreign investment (ln***fdi***), government intervention (***gov***), financial development (***fin***), industry structure (***is***) and education level (***edu***) (4–8). The definitions and descriptive statistics of each variable are shown in detail in Tables 1, 2. It should be noted that this paper measures urban education level through average years of education per capita (37). The specific algorithm is:


(9) $$\begin{array}{l} edu_{it}=6prim_{it}+9mid_{it}+12hig_{it}+16uni_{it} \end{array}$$

where *prim*_*it*_, *mid*_*it*_, *hig*_*it*_ and *uni*_*it*_ denote the proportion of residents with education level above elementary school, junior high school, high school and junior college in year *t* of city *i*.

**Table 1:** Variable definition.

**Classification**	**Symbol**	**Definition**	**Measurement**
Dependent variable	ln***mw***	Medical workforce	Logarithm of the total number of doctors in the city
	ln***mi***	Medical infrastructure	Logarithm of the total number of hospital beds in the city
Independent variables	BCCP	Broadband China city pilots	Takes the value of 1 if BCCP is implemented, 0 otherwise
Control variables	ln***rgdp***	Economic development	Logarithm of GDP per capita
	ln***pop***	Total population	Logarithm of population
	ln***fdi***	Foreign investment	Logarithm of foreign investment
	***gov***	Government intervention	Government expenditure/GDP
	***fin***	Financial development	Financial institution deposits/GDP
	***is***	Industry structure	Value-added ratio of tertiary industry
	***edu***	Education level	Average years of education per capita

**Table 2:** Descriptive statistics.

**Variable**	**Obs**	**Mean**	**Std**	**Min**	**Median**	**Max**
ln***mw***	2,590	8.975	0.725	6.804	8.949	11.659
ln***mi***	2,590	9.675	0.680	7.657	9.656	12.086
BCCP	2,590	0.196	0.397	0.000	0.000	1.000
ln***rgdp***	2,590	10.679	0.578	8.881	10.645	13.056
ln***pop***	2,590	5.936	0.641	3.922	5.958	8.134
ln***fdi***	2,590	10.093	1.852	1.099	10.174	14.941
***fin***	2,590	1.395	0.614	0.371	1.252	7.203
***gov***	2,590	0.187	0.085	0.044	0.168	1.485
***is***	2,590	40.573	9.888	14.360	39.675	83.520
***edu***	2,590	9.119	0.530	7.679	9.096	12.701
**Health care environment**	**Treated group**			**Control group**		
	**Pre-policy**	**Post-policy**	**Change**	**Pre-policy**	**Post-policy**	**Change**
ln***mw***	9.063	9.763	7.72%	8.396	8.722	3.88%
ln***mi***	9.823	10.687	8.80%	9.396	9.634	2.53%

## Results

### Spatial autocorrelation test

**Figure 2:**
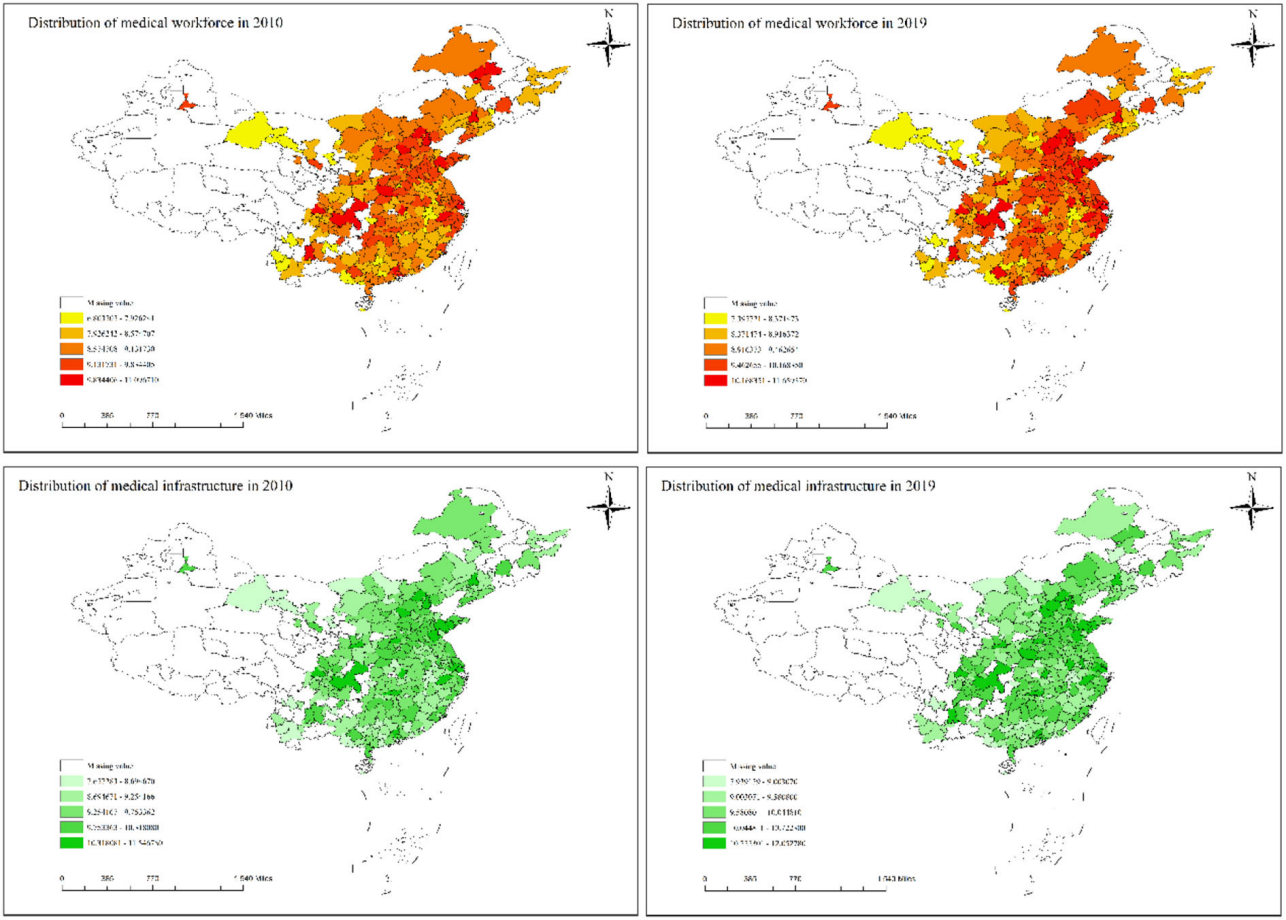
The spatial distribution of health care environment. The plots in the first row denote the spatial distribution of medical workforce (ln***mw***) in 2010 and 2019. The plots in the second row denote the spatial distribution of medical infrastructure (ln***mi***) in 2010 and 2019.

To analyze the spatial correlation of health care environment at the city level in China, this paper reports the spatial distribution of medical workforce and medical infrastructure through Figure 2. The spatial distribution in Figure 2 shows that medical workforce and medical infrastructure have relatively similar characteristics, i.e., the health care environment in the regional capital cities is stronger than that in the surrounding cities and shows a decreasing trend with distance. Meanwhile, the medical health environment in coastal areas and northern China is stronger than that in inland areas and southwest China. To further investigate the statistical significance of the spatial correlation of health care environment, this paper calculates the Moran's I index of medical workforce and medical infrastructure from 2010 to 2019.

**Figure 3:**
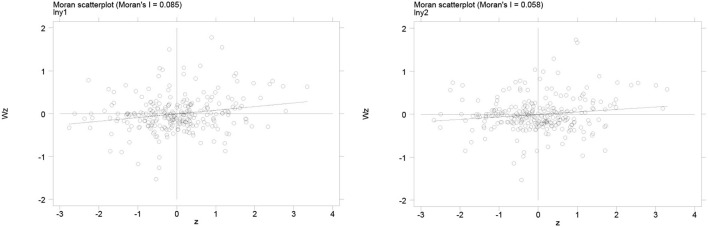
Moran scatterplot of health care environment in 2010. The left graph denotes the Moran scatterplot of medical workforce (ln***mw***) and the right graph denotes the Moran scatterplot of medical infrastructure (ln***mi***).

**Figure 4:**
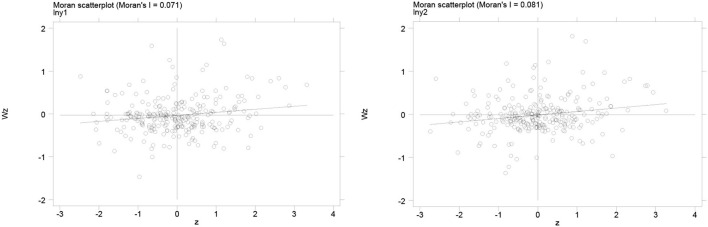
Moran scatterplot of health care environment in 2019. The left graph denotes the Moran scatterplot of medical workforce (ln***mw***) and the right graph denotes the Moran scatterplot of medical infrastructure (ln***mi***).

The Moran's I Index scatterplot provides a visual representation of the spatial correlation of urban healthcare environments. Figures 3, 4 shows the Moran scatter plots for the healthcare environment in 2010 and 2019. In these plots, the horizontal axis represents the standardized healthcare environment, and the vertical axis represents the spatial lagged values. According to the scatter plots, the coefficient of the main fit line is significantly higher than zero, indicating a spatially positive correlation between healthcare environments. The specific results of the Moran index of healthcare environment are given in Table 3. During 2010–2019, the Moran's I index is significantly positive at the 1% level with index values between 0 and 1, which indicates a strong spatial correlation of city-level health care environment in China. Therefore, the spatial factor should be considered in the estimation of the impact of information infrastructure development on health care environment.

**Table 3:** Calculation of Moran's I index of health care environment.

**Year**	**ln*mw***		**ln*mi***	
	**Moran's I**	**Z-value**	**Moran's I**	**Z-value**
2010	0.088^***^	2.871	0.060^**^	1.979
2011	0.053^**^	1.779	0.047^*^	1.578
2012	0.093^***^	3.035	0.060^**^	1.980
2013	0.078^***^	2.551	0.062^**^	2.069
2014	0.097^***^	3.142	0.063^**^	2.090
2015	0.099^***^	3.200	0.052^**^	1.738
2016	0.087^***^	2.840	0.056^**^	1.879
2017	0.089^***^	2.887	0.085^***^	2.764
2018	0.079^***^	2.578	0.083^***^	2.715
2019	0.073^***^	2.394	0.083^***^	2.720

***, **, and * denote significant at the 1, 5, and 10% level.

### Impact of information infrastructure development and health care environment

Table 4 reports the baseline regression results of the impact of information infrastructure development on health care environment. For comparison and to ensure the robustness of the results, we report all the results of the fixed effects model, the SLM model, the SEM model, and the SDM model. For each model, we report results with both medical workforce (ln***mw***) and medical infrastructure (ln***mi***) as dependent variables.

**Table 4:** Impact of information infrastructure development and health care environment.

**Variables**	**FE**		**SLM**		**SEM**		**SDM**	
	**lnmw**	**lnmi**	**lnmw**	**lnmi**	**lnmw**	**lnmi**	**lnmw**	**lnmi**
BCCP	0.075^***^	0.048^***^	0.037^***^	0.015^**^	0.036^***^	0.014^**^	0.039^***^	0.023^***^
ln**rgdp**	0.406^***^	0.367^***^	0.126^***^	0.090^***^	0.129^***^	0.096^***^	0.285^***^	0.208^***^
	(0.014)	(0.011)	(0.027)	(0.016)	(0.027)	(0.017)	(0.026)	(0.017)
ln**pop**	0.714^***^	0.572^***^	0.550^***^	0.471^***^	0.556^***^	0.481^***^	0.649^***^	0.527^***^
	(0.063)	(0.047)	(0.059)	(0.036)	(0.059)	(0.036)	(0.060)	(0.039)
ln**fdi**	−0.005	0.007^**^	0.005	0.004^*^	0.006	0.004^*^	0.000	0.004^*^
	(0.004)	(0.003)	(0.004)	(0.002)	(0.004)	(0.002)	(0.004)	(0.003)
**fin**	0.167^***^	0.111^***^	0.041^**^	0.007	0.042^**^	0.009	0.105^***^	0.045^***^
	(0.016)	(0.012)	(0.018)	(0.011)	(0.018)	(0.011)	(0.018)	(0.012)
**gov**	−0.048	0.342^***^	−0.282^***^	0.042	−0.276^***^	0.039	−0.156^*^	0.140^**^
	(0.088)	(0.066)	0.037	(0.051)	(0.083)	(0.051)	(0.085)	(0.055)
wBCCP							0.061^**^	0.044^***^
							(0.026)	(0.017)
wln**rgdp**							0.089^**^	−0.066^***^
							(0.037)	(0.024)
wln**pop**							−0.199	−0.541
							(0.155)	(0.100)
wln**fdi**							−0.045^***^	0.024^***^
							(0.010)	(0.006)
**wfin**							0.020	−0.044^*^
							(0.037)	(0.024)
**wgov**							0.005	0.520^***^
							(0.261)	(0.171)
City FE	Y	Y	Y	Y	Y	Y	Y	Y
Year FE	Y	Y	Y	Y	Y	Y	Y	Y
Obs	2590	2590	2590	2590	2590	2590	2590	2590
Log-L	342.74	472.33	1322.89	2586.61	1323.76	2589.97	1265.17	2334.54
R	0.469	0.810	0.817	0.854	0.549	0.820	0.822	0.593

The ***, **, and * symbols indicate the significant at the 1% level, 5% level, and 10% level respectively. City FE and Year FE denote the city fixed effects and year fixed effects.

According to the results, BCCP implementation has a significant positive contribution to the health care environment. The coefficients calculated by the four models for BCCP on medical workforce (ln***mw***) are 0.075 (*p* < 0.01), 0.037 (*p* < 0.01), 0.036 (*p* < 0.01), and 0.039 (*p* < 0.01), respectively. The coefficients for BCCP on medical infrastructure (ln***mi***) are 0.048 (*p* < 0.01), 0.015 (*p* < 0.01), 0.014 (*p* < 0.01), and 0.023 (*p* < 0.01). Since SDM takes into account both spatial lag effect and spatial error effect, its assessment of BCCP impact is more reliable. Therefore, the implementation of BCCP leads to a final improvement of 3.9 and 2.3% in medical workforce and medical infrastructure after excluding the spatial factor interference.

The estimation results of the SDM model suggest that the BCCP policy has an important enhancing effect on the health care environment. Since the cities of BCCP are distributed across the country and the health care environment is spatially relevant, it is necessary to discuss the spatial spillover effects. Table 5 further reports the spatial spillover effects of BCCP on the health care environment. Specifically, the direct effects, indirect effects, and total effects of BCCP implementation on medical workforce (ln***mw***) are 4.1, 7.2, and 11.3%, which all passed the 1% significance test. The direct effects, indirect effects, and total effects of BCCP implementation on medical infrastructure (ln***mi***) are 2.9, 12.5, and 15.4%, which also passed the 1% significance test. The results of the above analysis show that information infrastructure development not only leads to significant improvements in the local healthcare environment, but also generates stronger positive spillover effects. The implementation of BBCP leads to an overall improvement effect of 11.3 and 15.4% for medical workforce and medical infrastructure nationwide. This shows that there is a need for the government to further promote the development of information infrastructure, mainly 5G, cloud computing and big data centers.

**Table 5:** Direct effect, indirect effect, and total effect of SDM in Table 4.

		**BCCP**	**ln*rgdp***	**ln*pop***	**ln*fdi***	***fin***	***gov***
Direct	ln***mw***	0.041^***^	0.287^***^	0.653^***^	−0.001	0.106^***^	−0.152^*^
effect		(0.012)	(0.025)	(0.058)	(0.004)	(0.018)	(0.084)
	ln***mi***	0.029^***^	0.212^***^	0.510^***^	0.007^***^	0.043^***^	0.199^***^
		(0.008)	(0.016)	(0.040)	(0.003)	(0.012)	(0.060)
Indirect	ln***mw***	0.072^**^	0.143^***^	−0.105	−0.049^***^	0.038	0.006
effect		(0.028)	(0.034)	(0.168)	(0.010)	(0.040)	(0.299)
	ln***mi***	0.125^***^	0.115^***^	−0.500^**^	0.060^***^	−0.041	1.350^***^
		(0.035)	(0.036)	(0.222)	(0.013)	(0.052)	(0.387)
Total	ln***mw***	0.113^***^	0.429^***^	0.548^***^	−0.049^***^	0.144^***^	−0.146
effect		(0.028)	(0.027)	(0.182)	(0.011)	(0.045)	(0.322)
	ln***mi***	0.154^***^	0.326^***^	0.010	0.066^***^	0.002	1.549^***^
		(0.037)	(0.036)	(0.242)	(0.014)	(0.058)	(0.419)

***, **, and * denote significant at the 1, 5, and 10% level.

### Parallel trend test

**Figure 5:**
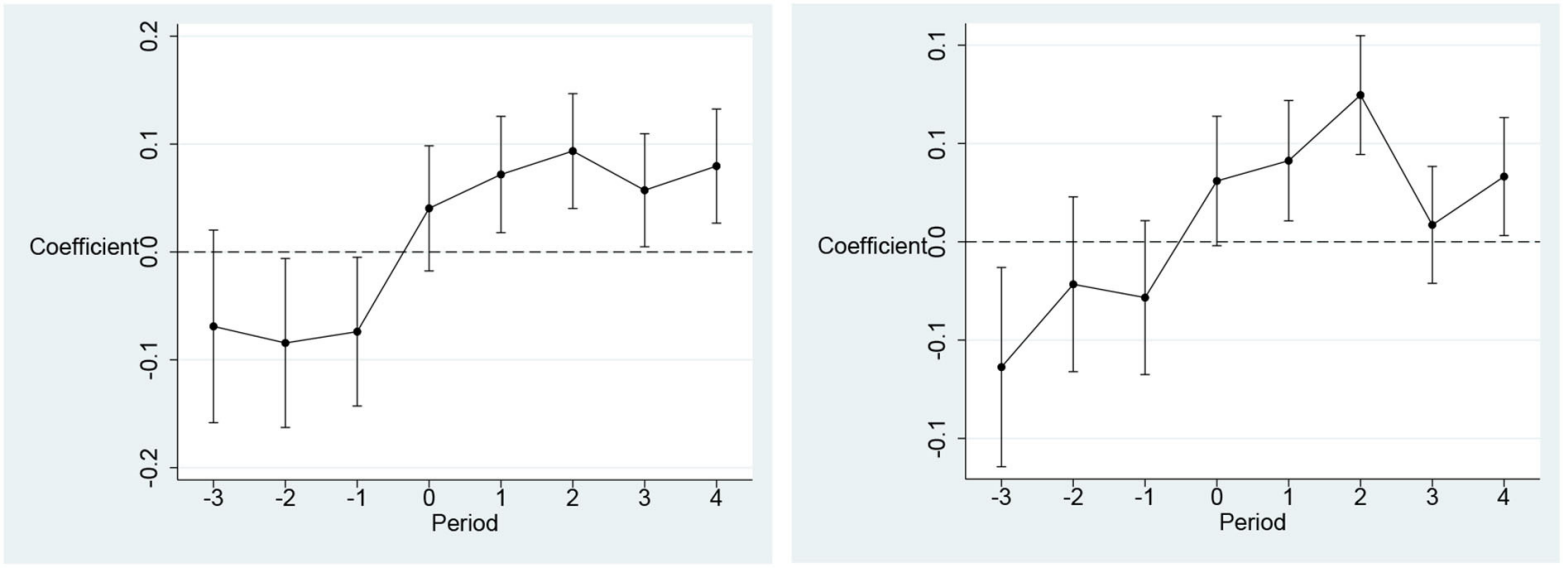
Results of Parallel Trend Test. The dependent variable in the left panel is medical workforce (ln***mw***) and the dependent variable in the right panel is medical infrastructure (ln***mi***). The X-axis denotes the window period for BCCP implementation. The Y axis represents the regression coefficient of BCCP. The year before BCCP is implemented as the base period.

Figure 5 reports the results of the parallel trend test. The regression coefficients mostly failed the 5% level significance test before the implementation of the BCCP. This indicates that there was no significant difference of the health care environment between the control groups and treated groups before the BCCP was implemented. The parallel trend hypothesis was satisfied. In addition, the regression coefficients showed a trend of increasing and then decreasing after the implementation of the BCCP. This indicates that the effect is strongest in the first 3 years of information infrastructure development. Over time, the impact of BCCP on the health care environment begins to decline. This implies that in the short term, BCCP can lead to an improvement in the health care environment, but the effect will gradually diminish. The government should ensure the short-term performance of the policy while increasing the long-term effectiveness of information infrastructure development on the effect of healthcare environment improvement.

### Analysis of the mechanisms by which information infrastructure development affects the health care environment

To test the mechanism of the effect of information infrastructure development affecting the health care environment in hypothesis 1, the mediating effect model is constructed in section Mediating effect model. Table 6 reports the regression results for this model.

**Table 6:** Mechanisms of information infrastructure affects the health care environment.

**Variables**	***iu***	**ln*mw***	**ln*mi***	***edu***	**ln*mw***	**ln*mi***
BCCP	0.085^***^	0.057^***^	0.051^***^	0.251^***^	0.078^***^	0.056^***^
	(0.003)	(0.012)	(0.009)	(0.012)	(0.016)	(0.009)
***iu***		0.612^***^	0.315^***^			
		(0.093)	(0.070)			
***edu***					0.085^***^	0.047^***^
					(0.031)	(0.018)
C	0.389^***^	0.797^**^	1.979^***^	9.065^***^	3.136^***^	3.147^***^
	(0.001)	(0.386)	(0.291)	(0.005)	(0.751)	(0.312)
Control	Y	Y	Y	Y	Y	Y
City FE	Y	Y	Y	Y	Y	Y
Year FE	Y	Y	Y	Y	Y	Y
Obs	2,590	2,590	2,590	2,590	2,590	2,590
F-static	714.181	305.227	515.698	406.731	52.750	216.989
Adj-R^2^	0.150	0.420	0.564	0.164	0.224	0.424

(1) *** and ** denote significant at the 1 and 5% level. (2) City FE and Year FE denote the city fixed effects and year fixed effects.

According to the regression results in columns (1) and (4), the regression coefficients of BCCP are 0.085 (*p* < 0.01) and 0.251 (*p* < 0.01), which means that the development of information infrastructure significantly promotes the industrial upgrading and the level of education in the city where it is located. Subsequently, the regression results in columns (2) and (3), the regression coefficients of ***iu*** are 0.612 (*p* < 0.01) and 0.315 (*p* > 0.1), respectively. This indicates that information infrastructure development enhances the healthcare environment by promoting industrial upgrading. The regression coefficients for ***edu*** in columns (5) and (6) are 0.085 (*p* < 0.05) and 0.047 (*p* < 0.05), respectively. This indicates that information infrastructure development enhances the health care environment by promoting the level of access to education.

### Heterogeneity analysis

Table 7 reports the results of the heterogeneity analysis of the implementation of BCCP on the health care environment. From the regional perspective, this paper analyzes the impact differences between the central and western regions relative to the eastern region. The results show that the enhancement of BCCP on medical workforce (ln***mw***) is mainly concentrated in the eastern region, but the enhancement of medical infrastructure (ln***mi***) is mainly concentrated in the central region and the western region.

**Table 7:** Heterogeneity analysis.

**Variables**	**Region**		**Administrative rank of city**	
	**ln*mw***	**ln*mi***	**ln*mw***	**ln*mi***
BCCP	0.045^**^	0.012	0.019^***^	0.013^***^
	(0.018)	(0.011)	(0.003)	(0.002)
BCCP × Central	−0.025	0.028^**^		
	(0.022)	(0.014)		
BCCP × Western	−0.034	0.040^***^		
	(0.024)	(0.015)		
BCCP × Rank			0.042^*^	0.048^***^
			(0.022)	(0.013)
ln***rgdp***	0.161^***^	0.097^***^	0.151^***^	0.090^***^
	(0.029)	(0.017)	(0.028)	(0.017)
ln***pop***	0.512^***^	0.457^***^	0.516^***^	0.442^***^
	(0.059)	(0.036)	(0.060)	(0.036)
ln***fdi***	0.007^*^	0.004^*^	0.007^*^	0.004^*^
	(0.004)	(0.002)	(0.004)	(0.002)
***fin***	0.057^***^	0.014	0.053^***^	0.007
	(0.019)	(0.012)	(0.019)	(0.012)
***gov***	−0.253^***^	0.051	−0.253^***^	0.043
	(0.083)	(0.051)	(0.083)	(0.051)
***w***BCCP	0.107^**^	0.063^**^	0.006	0.041^*^
	(0.043)	(0.026)	(0.036)	(0.022)
***w***BCCP × Central	−0.134^**^	−0.085^**^		
	(0.064)	(0.039)		
***w***BCCP × Western	−0.114^*^	−0.019		
	(0.063)	(0.038)		
***w***BCCP × Rank			0.057	−0.075^**^
			(0.054)	(0.033)
***w***ln***rgdp***	−0.135^***^	−0.124^***^	−0.145^***^	−0.126^***^
	(0.045)	(0.028)	(0.044)	(0.027)
***w***ln***pop***	−0.548^***^	−0.378^***^	−0.509^***^	−0.302^***^
	(0.156)	(0.097)	(0.157)	(0.097)
***w***ln***fdi***	−0.017^*^	0.005	−0.017	0.005
	(0.010)	(0.006)	(0.010)	(0.006)
***wfin***	−0.059	−0.048^*^	−0.051	−0.037
	(0.041)	(0.025)	(0.040)	(0.025)
***wgov***	−0.393	0.275^*^	−0.395	0.279^*^
	(0.266)	(0.163)	(0.266)	(0.163)
City FE	Y	Y	Y	Y
Year FE	Y	Y	Y	Y
Obs	2,590	2,590	2,590	2,590
*R* ^2^	0.522	0.551	0.525	0.574

(1) ***, **, and * denote significant at the 1, 5, and 10% level. (2) City FE and Year FE denote the city fixed effects and year fixed effects. (3) For ordinary prefecture-level cities, the value of rank is equal to 1; for non-ordinary prefecture-level cities, rank takes the value of 0.

Specifically, the effect of BCCP on the improvement of medical workforce in the eastern region is 4.5%, and there is no significant difference between the improvement effect of the eastern region on the central region and the western region. Compared to the eastern region, the effect of BCCP on medical infrastructure is 2.8 and 4% higher in the central and western region. In addition, this paper further analyzes the impact of BCCP on cities with different administrative ranks. In this paper, provincial capital cities, sub-provincial cities and municipalities are defined as non-ordinary prefecture-level cities, and other cities are ordinary prefecture-level cities. The results show that BCCP enhances the health care environment significantly less in ordinary prefecture-level cities than in non-ordinary prefecture-level cities. Specifically, the BCCP has a 4.2 and 4.8% higher lift effect on medical workforce and medical infrastructure for non-average prefectures than for average prefectures.

### Robustness test

#### Placebo test

**Figure 6:**
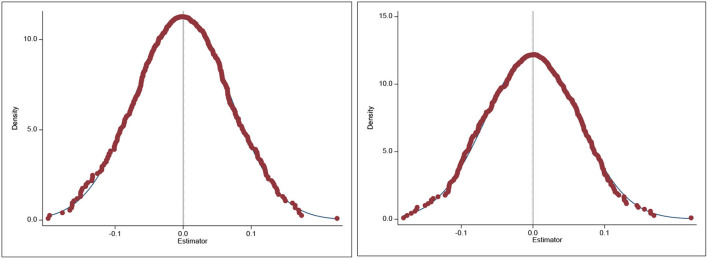
Results of placebo test. The dependent variable in the left graph is medical workforce (ln***mw***) and the dependent variable in the right graph is medical infrastructure (ln***mi***). Treatment groups were randomly drawn 500 times in the control group by Monte Carlo simulation and DID regression was performed. Plot the obtained regression coefficients as a distribution graph. This figure reports the results of health care environment of non-pilot cities as a dependent variable, presenting a normal distribution with an average value of 0.

Since the BCCP policy may also affect the health care environment in non-pilot cities, this would lead to unreliable estimates. This study adopts Monte Carlo simulation to conduct a placebo test according to the study of La Ferrara et al. (38). First, this study randomly selected samples from the control group multiple times as the treatment group. Then, this study performs DID regression analysis to estimate the parameters. The results are reliable if the estimated parameters are normally distributed with a mean value of 0. The estimated coefficient distributions and kernel density curves after 500 random samples are given in Figure 6. As expected from the placebo test, the estimated coefficients show a normal distribution with a mean around 0. This suggests that the change in the health care environment of the truly treated group stems from the implementation of BCCP policy.

#### Re-estimation using PSM-DID

To enhance the robustness of the study, this paper uses both PSM methods for posterior matching regression. This paper used two conventional matching methods, namely 1:1 nearest neighbor matching and kernel density matching.

The regression results after matching using these two methods are reported in Table 8. The regression results include both estimates with medical workforce and medical infrastructure as dependent variables. The results show that the implementation of BCCP significantly improves the urban health care environment using either nearest neighbor matching or kernel density matching. This is consistent with the results of previous studies using spatial panels, i.e., the results of this paper are robust.

**Table 8:** Re-estimation using PSM-DID.

**Variables**	**Neighbor matching**		**Kernel matching**	
	**(*****n*** **=** **1)**			
	**ln*mw***	**ln*mi***	**ln*mw***	**ln*mi***
BCCP	0.109^***^	0.125^***^	0.083^***^	0.087^***^
	(0.017)	(0.015)	(0.020)	(0.014)
ln***rgdp***	0.437^***^	0.150^***^	0.435^***^	0.384^***^
	(0.028)	(0.024)	(0.017)	(0.012)
ln***pop***	0.764^***^	0.727^***^	0.736^***^	0.465^***^
	(0.098)	(0.087)	(0.088)	(0.064)
ln***fdi***	−0.013^**^	0.006	−0.004	0.006
	(0.006)	(0.005)	(0.005)	(0.003)
***fin***	0.133^***^	0.015	0.192^***^	0.120^***^
	(0.022)	(0.019)	(0.020)	(0.014)
***gov***	0.051	0.085	−0.126	0.273^***^
	(0.113)	(0.100)	(0.099)	(0.072)
C	−0.285	3.741^***^	−0.249	2.526^***^
	(0.600)	(0.533)	(0.505)	(0.364)
City FE	Y	Y	Y	Y
Year FE	Y	Y	Y	Y
Obs	866	866	1,898	1,898
F-static	123.548	61.706	239.944	345.291
Adj-*R*^2^	0.374	0.145	0.396	0.497

(1) *** and ** denote significant at the 1 and 5% level. (2) City FE and Year FE denote the city fixed effects and year fixed effects.

## Conclusion and recommendations

### Conclusion

This paper adopts a spatial DID model to assess the impact of information infrastructure development on the health care environment based on a quasi-natural experiment of BCCP. First, the spatial distribution map of the health care environment and the results of the Moran's I index measure indicate that there is a significant spatial correlation between medical workforce and medical infrastructure. The health care environment in north region and coastal areas is higher than that in the central and western regions in China.

Second, the estimation results of the spatial DID indicated that the implementation of BCCP improved medical workforce and medical infrastructure by 4.1 and 2.9%, respectively. In addition, BCCP implementation improved medical workforce and medical infrastructure by 7.2 and 12.5% in the surrounding areas. The total effects of BCCP implementation on medical workforce and medical infrastructure improvement are 11.3 and 15.4%. The results suggest that information infrastructure development not only significantly improves the local health care environment, but also has positive spatial spillover effects.

Third, the results of the mechanism analysis suggest that information infrastructure development enhances the health care environment by promoting industrial upgrading and education level. This paper also analyzes the heterogeneity of cities in different regions and administrative levels. The results show that BCCP has a higher impact on the eastern region and non-ordinary prefecture-level cities of medical workforce. Meanwhile, the impact of BCCP is higher for medical infrastructure in central and western regions with non-ordinary prefecture-level cities.

Finally, this paper also performs a series of robustness tests to ensure the reliability of the analysis results. Parallel trend test showed that there was no significant difference in health care environment between the treated groups and control groups before the implementation of BCCP, while the health care environment of the treated group is significantly higher than that of the control group after the implementation of BCCP. In addition, placebo tests, and re-estimates based on PSM-DID all yielded more consistent conclusions. Thus, the assessment of spatial DID was relatively reliable.

### Recommendations

Based on the conclusions, this paper puts forward the following three recommendations.

First, it is necessary to continuously promote the development of information infrastructure. Digitization and information technology are important tools to improve the regional health care environment. Especially for developing regions, their ability to absorb excellent medical resources is more limited. The development of information technology can enhance the health care environment of such regions through inter-regional medical cooperation. The results of this paper show that information infrastructure development not only enhances the local healthcare environment, but also has a spillover effect on the surrounding areas. This implies that there is no strong competition among cities, and that cooperation and sharing can maximize the regional healthcare environment. The government can develop a larger-scale information infrastructure development program from a regional perspective. For other developing countries, information infrastructure development policies like that of China could be developed. IT development driven by initial government investment, thus improving the healthcare environment in the country.

Second, the government should encourage the combination of information technology industry and traditional medical industry. Cutting-edge information technology and data analysis techniques can greatly enhance the efficiency of medical personnel and reduce their errors of judgment. At the same time, the government should also encourage healthcare professionals to learn about information technology to further enhance the healthcare environment. For both developed and developing countries, governments should encourage the integration of IT with the healthcare industry through channels such as subsidies from the Ministry of Finance or innovation support. Information technology development can effectively bridge the medical gap between regions and improve the medical and health care environment.

Finally, part of governments should enhance the improvement of health care environment by the means of financial subsidies for information infrastructure development. The findings of this paper suggest that the impact of information infrastructure development on western regions and ordinary prefecture-level cities is more limited. To address this phenomenon, local governments should increase their initial investment and build healthcare platforms through financial subsidies. Meanwhile, governments should make full use of the industrial upgrading and education level improvement effects brought by the information infrastructure development to improve the medical and health care environment at a later stage.

## Data availability statement

Publicly available datasets were analyzed in this study. This data can be found here: The data are obtained from the China City Statistics Database (CCSD) in the China Research Data Service (CNRDS) platform (https://www.cnrds.com/Home/Index#/FinanceDatabase/DB/CCSD).

## Author contributions

CT: conceptualization, data curation, methodology, and writing—original draft. CZ: writing—review and editing and software. CY: funding acquisition, supervision, validation, and project administration. YT and YZ: writing—review and editing. All authors contributed to the article and approved the submitted version.

## Funding

This work was supported by National Social Science Fund of China (Grant No. 19CGL008).

## Conflict of interest

The authors declare that the research was conducted in the absence of any commercial or financial relationships that could be construed as a potential conflict of interest.

## Publisher's note

All claims expressed in this article are solely those of the authors and do not necessarily represent those of their affiliated organizations, or those of the publisher, the editors and the reviewers. Any product that may be evaluated in this article, or claim that may be made by its manufacturer, is not guaranteed or endorsed by the publisher.
